# Exosomes Derived from Adipose Mesenhymal Stem Cells Ameliorate Lipid Metabolism Disturbances Following Liver Ischemia-Reperfusion Injury in Miniature Swine

**DOI:** 10.3390/ijms252313069

**Published:** 2024-12-05

**Authors:** Xiangyu Lu, Yue Wang, Chenxi Piao, Pujun Li, Lei Cao, Tao Liu, Yajun Ma, Hongbin Wang

**Affiliations:** 1 College of Veterinary Medicine, Northeast Agricultural University, Harbin 150030, China; lxy1997202206@163.com (X.L.); pcx7001@163.com (C.P.); pujunli1024@163.com (P.L.); caolei_001010@163.com (L.C.); liutaotiger@163.com (T.L.); mayajun1994@126.com (Y.M.); 2 Heilongjiang Provincial Key Laboratory of Pathogenic Mechanism for Animal Disease and Comparative Medicine, Harbin 150030, China; wangyuemooney@163.com; 3 College of Animal Science and Technology, Henan University of Science and Technology, Luoyang 471000, China

**Keywords:** hepatectomy, ischemia-reperfusion injury, adipose mesenchymal stem cell-derived exosomes, lipid metabolism, miniature swine

## Abstract

The liver plays a crucial role in regulating lipid metabolism. Our study examined the impact of Exosomes derived from adipose mesenchymal stem cells (ADSCs-Exo) on lipid metabolism following liver ischemia-reperfusion injury (IRI) combined with partial hepatectomy. We developed a miniature swine model for a minimally invasive hemi-hepatectomy combined with liver IRI. In this study, we administered PBS, ADSCs-Exo, and adipose-derived stem cells (ADSCs) individually through the portal vein. Before and after surgery, we evaluated various factors including hepatocyte ultrastructure, lipid accumulation in liver tissue, and expression levels of genes and proteins associated with lipid metabolism. In addition, we measured serum and liver tissue levels of high-density lipoprotein (HDL), low-density lipoprotein (LDL), triglycerides (TG), and total cholesterol (CHOL). TEM and oil red O stain indicated a significant reduction in liver steatosis following ADSCs-Exo treatment, which also elevated serum levels of HDL, LDL, TG, and CHOL. Additionally, ADSCs-Exo have been shown to significantly decrease serum concentrations of HDL, LDL, TG, and CHOL in the liver (*p* < 0.05). Finally, ADSCs-Exo significantly downregulated lipid synthesis-related genes and proteins, including SREBP-1, SREBP-2, ACC1, and FASN (*p* < 0.05), while upregulating lipid catabolism-related genes and proteins, such as PPAR-α and ACOX1 (*p* < 0.05). ADSCs-Exo as a cell-free therapy highlights its therapeutic potential in hepatic lipid metabolism abnormalities.

## 1. Introduction

Liver ischemia-reperfusion injury (IRI) is a common complication of hepatic resection, liver transplantation, and hemorrhagic shock, and is the result of the reestablishment of blood flow to hepatic tissues after the interruption. When liver IRI occurs, it can cause an acute inflammatory response, leading to significant hepatocellular injury and liver dysfunction, and even multi-organ failure. Since the concept of IRI was first introduced in the 1960s, no effective drugs have been developed for clinical intervention [1,2].

It is well known that the liver is an important regulator of metabolic homeostasis and is involved in metabolic activities such as lipid and glucose metabolism. In the event of liver damage, the organ’s metabolic function becomes particularly vulnerable to irregularities, which can ultimately result in the development of metabolic disorders [3]. Previous studies have shown that when IRI occurs in the liver, a large amount of abnormal lipid accumulation is found in the liver [4], suggesting that lipid metabolism disorders develop following liver IRI. This suggests that lipid metabolic signaling may play a key role in triggering ischemia-reperfusion-induced hepatocyte injury, providing an interesting link between metabolism, inflammation, and liver IRI [5]. Lipid metabolism includes lipogenesis, lipolysis, and lipid oxidation, and the liver is a crucial organ for lipid metabolism and the maintenance of lipid homeostasis [6]. Normal lipid metabolism in the liver consists of four main processes: lipid synthesis, uptake, efflux, and oxidation. Hepatocyte mitochondria, peroxisomes, and mitochondrial β-oxidation are involved in lipid metabolism [7]. Peroxisome proliferator-activated receptor alpha (PPARα) is a pivotal transcription factor for fatty acid (FA) beta-oxidation. Upon entering hepatocytes, triglycerides (TG) and FA stimulate mitochondrial β-oxidation, which in turn produces ATP through the modulation of its downstream targets, including carnitine palmitoyltransferase 1A (CPT1A), carnitine octanoyltransferase (CROT), and Acyl-CoA oxidase 1 (ACOX1), by peroxisome proliferator-activated receptor alpha (PPARα). The remaining portion is stored as lipid droplets in hepatocytes in the presence of stearoyl coenzyme A desaturase 1 (SCD1), acetyl coenzyme A carboxylase (ACC), and fatty acid synthase (FASN) [8,9]. Furthermore, sterol regulatory element binding protein 1 (SREBP-1) and sterol regulatory element binding protein 2 (SREBP-2) are crucial regulators of lipid and glucose metabolism, exerting significant influence on the stimulation of lipogenesis, gluconeogenesis, and the pentose phosphate pathway [9]. The principal factors associated with lipid metabolism interact in a harmonious manner to sustain the dynamic equilibrium of lipid metabolism in hepatic tissues. This process can markedly enhance the recuperation of liver function and mitigate the severity of liver injury [10]. Thus, rectifying the disruption of lipid metabolism following liver IRI is a key factor in mitigating liver damage.

Mesenchymal stem cells (MSCs) are pluripotent cells with the capacity to differentiate into various different mesenchymal cell lines. The capacity for self-renewal, multispectral differentiation potential, paracrine effects, and immunomodulatory properties render this a promising candidate for research in regenerative medicine. MSCs are used in the treatment of alveolar ridge fracture defects, jaw defect reconstruction, and maxillary sinus augmentation with good results [11,12,13]. The role of MSCs in attenuating myocardial injury was first reported in 2002 and purified bone marrow mesenchymal stem cells transplanted into the mouse heart appeared to differentiate into cardiomyocytes [14]. Adipose-derived mesenchymal stem cells (ADSCs) are among the most commonly utilized types of MSCs in research due to their convenient accessibility, high yield [15], rapid proliferation [16], and low immunogenicity [17]. Recent studies have shown that MSCs may not act directly, but rather through the secretion of Exosomes (Exo), which are usually between 30 and 150 nanometers in diameter [18]. In addition, Exo may have advantages in immunomodulation, anti-inflammatory functions, and tissue regeneration compared to parental MSCs [19]. Therefore, Exosomes derived from MSCs (MSCs-Exo) are increasingly recognized as effective non-cellular substitutes for tissue repair and regeneration [20]. Previous studies have demonstrated the protective effects of MSCs-Exo against IRI in various tissues, including the brain [21], myocardium [22], kidney [23], and liver [24]. In the context of lipid metabolism, Exo participation encompasses a range of processes, including lipid synthesis, transport, and degradation. This is achieved through the transfer of a variety of molecules, including fatty acids, cholesterol, ceramides, genetic material, and enzymes [25]. ADSCs-Exo has been demonstrated to enhance insulin sensitivity, mitigate obesity, and alleviate hepatic steatosis in a murine model of obesity [26]. ADSCs-Exo has also been demonstrated to delay the progression of non-alcoholic fatty liver disease (NAFLD) by the delivery of the anti-fibrotic microRNA-122 [27]. Despite the plethora of studies that have demonstrated the beneficial effects of MSCs-Exo on lipid metabolism and hepatic injury, the majority of these studies have focused on NAFLD. Consequently, there is a paucity of knowledge regarding the abnormalities of lipid metabolism caused by liver IRI. Additionally, most research has concentrated on small animal models, with fewer studies involving large animals. This limitation hinders the transition from animal research to human medicine.

Appropriate animal models are important in scientific research. Rodents are commonly used as model animals [28], but due to genetic and anatomical differences, they are not fully able to respond to human pathophysiological features, hindering the progress of research towards human medicine. In recent years, studies have shown that miniature swine are more similar to humans in physiology, anatomy, nutrient metabolism, drug metabolism, and disease development [29,30], which has greatly facilitated the study of human diseases.

Therefore, this study employed laparoscopic, minimally invasive techniques to induce liver IRI combined with hemi-hepatectomy in miniature swine, followed by treatment with ADSCs-Exo. The experiment aimed to explore the effects of ADSCs-Exo on lipid metabolism abnormalities after liver IRI. To provide insight into the use of MSCs-Exo for the treatment of human liver injury from a comparative medicine perspective.

## 2. Results

### 2.1. Characteristics of ADSCs and ADSCs-Exo

The results discussed in this section are covered in our previous publication by the research team [31], specifically in Sections 3.1 and 3.2. No further elaboration is necessary at this point.

### 2.2. Ultrastructural Analysis of Hepatocytes

**Figure 1:**
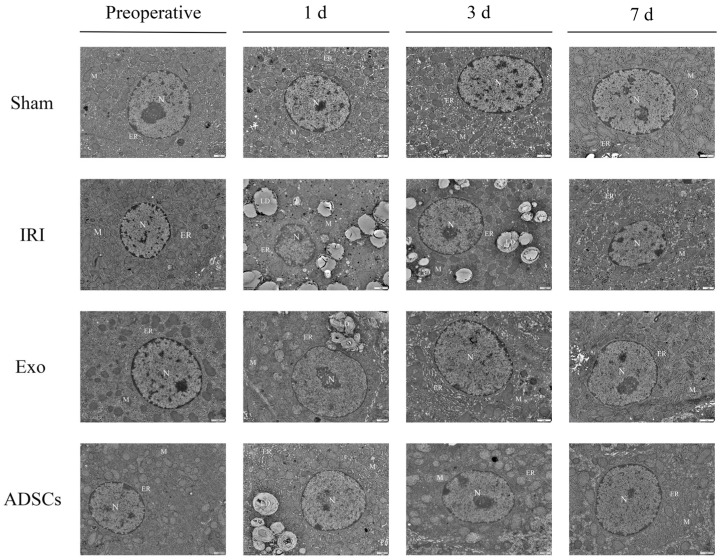
Ultrastructure of liver tissue observed by TEM (15,000×). **N** represents nucleus, **M** represents mitochondria, **ER** represents endoplasmic reticulum and **LD** represents lipid droplets.

Figure 1 presents the results of the ultrastructural analysis of liver cells in each group. Before surgery, all groups showed no structural abnormalities in hepatocytes. One day after surgery, hepatocyte structure was normal in the Sham group. However, the IRI group exhibited a large accumulation of lipid droplets, while the Exo and ADSCs groups showed a small accumulation. Three days after surgery, hepatocyte ultrastructure remained normal in the Sham group. The IRI group still exhibited lipid droplet accumulation, while the Exo and ADSCs groups showed no accumulation. Seven days after surgery, hepatocytes in all groups displayed normal structure.

### 2.3. ADSCs-Exo Reduce Hepatic Lipid Accumulation

**Figure 2:**
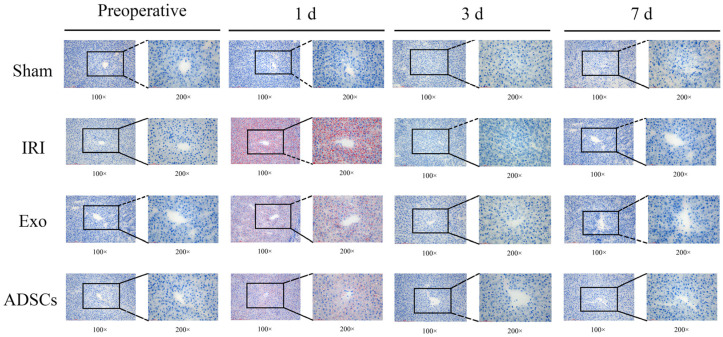
Oil red O staining in different groups.

We studied the impact of ADSCs-Exo on hepatic lipid metabolism after hepatic IRI and partial resection. This was conducted by measuring lipid accumulation in miniature swine using oil red O staining. One day after surgery, we observed significant lipid accumulation in the IRI group. In contrast, both the Exo and ADSCs groups showed reduced lipid accumulation compared to the IRI group. No lipid accumulation was detected in any of the groups at subsequent points in time. These findings are illustrated in Figure 2.

### 2.4. Results of HDL, LDL, TG, and CHOL in Serum

**Figure 3:**
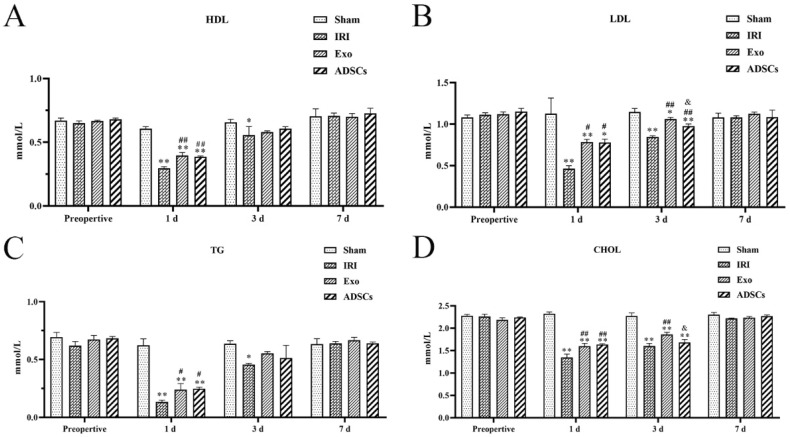
Effect of ADSCs-Exo on HDL (**A**), LDL (**B**), TG (**C**), and CHOL (**D**) levels in serum. Results are expressed as mean ± SD, * 0.01 < *p* < 0.05, ** *p* < 0.01 versus the Sham group. ^#^ 0.01 < *p* < 0.05, ^##^ *p* < 0.01 versus the IRI group. ^&^ 0.01 < *p* < 0.05, versus the ADSCs-Exo group.

We assessed the modulatory effects of Exo and ADSCs on serum levels of HDL, LDL, TG, and CHOL after IRI of the right hemisphere combined with a resection of the left hemisphere. The results are shown in Figure 3. At one day after surgery, HDL (Figure 3A), LDL (Figure 3B), TG (Figure 3C), and CHOL (Figure 3D) levels were significantly lower in the IRI group, the Exo group, and the ADSCs group compared with the Sham group (*p* < 0.01 or 0.01 < *p* < 0.05), and the levels of HDL, LDL, TG, and CHOL were found to be significantly higher in the Exo and ADSCs groups compared with the IRI group (*p* < 0.01 or 0.01 < *p* < 0.05). At three days after surgery, the levels of HDL, LDL, TG, and CHOL were found to be significantly lower in the IRI group compared with the Sham group (*p* < 0.01 or 0.01 < *p* < 0.05), and the levels of LDL and CHOL were found to be significantly lower in the Exo and ADSCs groups (*p* < 0.01 or 0.01 < *p* < 0.05), and the levels of LDL were found to be significantly higher in both the Exo and ADSCs groups (*p* < 0.01), while the levels of CHOL were found to be significantly higher in the Exo group (*p* < 0.01) in comparison to the IRI group. Nevertheless, the levels of LDL and CHOL were demonstrably lower in the ADSCs group in comparison to the Exo group (0.01 < *p* < 0.05). There were no significant differences in HDL, LDL, TG, and CHOL levels between the preoperative and 7 d postoperative groups.

### 2.5. Results of HDL, LDL, TG, and CHOL in Liver Tissue

**Figure 4:**
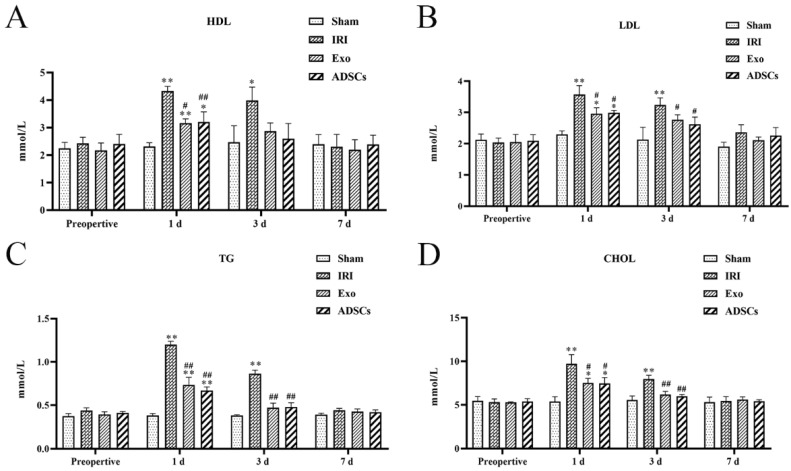
Effect of ADSCs-Exo on HDL (**A**), LDL (**B**), TG (**C**), and CHOL (**D**) levels in liver tissue. Results are expressed as mean ± SD, * 0.01 < *p* < 0.05, ** *p* < 0.01 versus the Sham group. ^#^ 0.01 < *p* < 0.05, ^##^ *p* < 0.01 versus the IRI group.

We assessed the modulatory effects of Exo and ADSCs on HDL, LDL, TG, and CHOL content in liver tissue after IRI of the right hemisphere combined with a hepatic resection of the left hemisphere. The results are shown in Figure 4. At one day after surgery, the levels of HDL (Figure 4A), LDL (Figure 4B), TG (Figure 4C), and CHOL (Figure 4D) were significantly higher in the IRI and Exo groups as well as in the ADSCs group compared with the Sham group (*p* < 0.01 or 0.01 < *p* < 0.05), and the levels of HDL, LDL, TG, and CHOL were found to be significantly lower in the Exo and ADSCs groups compared with the IRI group (*p* < 0.01 or 0.01 < *p* < 0.05). At three days after surgery, the levels of HDL, LDL, TG, and CHOL were found to be significantly higher in the IRI group compared to the Sham group (*p* < 0.01 or 0.01 < *p* < 0.05), and the levels of LDL, TG, and CHOL were found to be significantly lower in the Exo and ADSCs groups compared to the IRI group. No significant differences were observed in HDL, LDL, TG, and CHOL levels between the preoperative and 7-d postoperative groups.

### 2.6. Effect of ADSCs-Exo on the Expression of Genes Related to Lipid Metabolism

**Figure 5:**
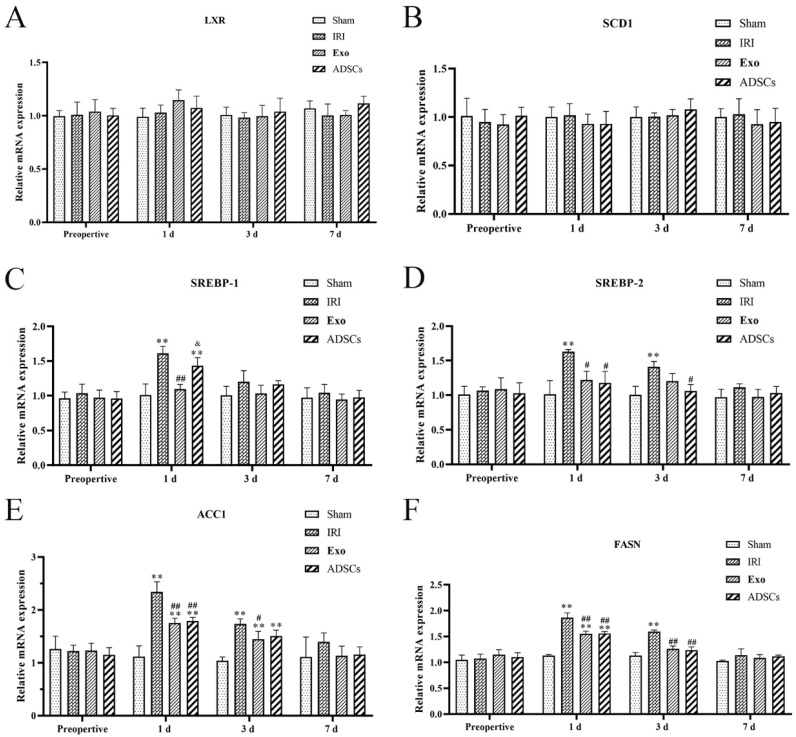
The mRNA expression of LXR (**A**), SCD1 (**B**), SREBP-1 (**C**), SREBP-2 (**D**), ACC1 (**E**), and FASN (**F**) in the liver. Results are presented as mean ± SD, ** *p* < 0.01 versus the Sham group. ^#^ 0.01 < *p* < 0.05, ^##^ *p* < 0.01 versus the IRI group. ^&^ 0.01 < *p* < 0.05.

The lipid synthesis gene expression results are shown in Figure 5. The mRNA expression levels of LXR and SCD1 (Figure 5A,B) did not differ significantly among the groups at any point in time. The mRNA expression levels of the SREBP-1, SREBP-2, ACC1 and FASN (Figure 5C–F) were extremely significantly higher in the IRI group on days post-surgery compared to that in the Sham group (*p* < 0.01), while they were downregulated by ADSCs-Exo and ADSCs (*p* < 0.01 or 0.01 < *p* < 0.05 versus IRI). The mRNA expression levels of the SREBP-2, ACC1, and FASN (Figure 5D–F) were extremely significantly higher in the IRI group three days post-surgery compared to that in the Sham group (*p* < 0.01), while they were downregulated by ADSCs-Exo and ADSCs (*p* < 0.01 or 0.01 < *p* < 0.05 versus IRI), and ADSCs downregulated the expression of SREBP-1 mRNA(0.01 < *p* < 0.05, versus IRI). There were no significant differences in the mRNA expression levels of SREBP-1, SREBP-2, ACC1, and FASN at other points in time.

**Figure 6:**
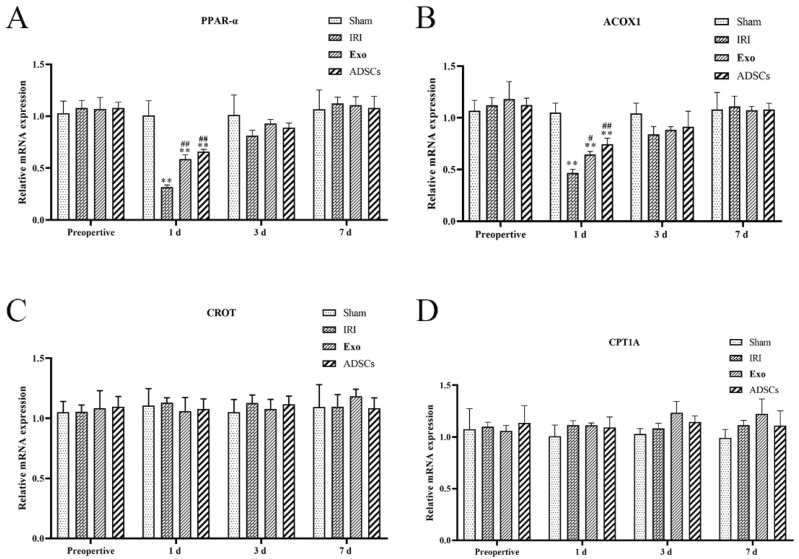
The mRNA expression of PPAR-α (**A**), ACOX1 (**B**), CROT (**C**), and CPT1A (**D**) in the liver. Results are presented as mean ± SD, ** *p* < 0.01 versus the Sham group. ^#^ 0.01 < *p* < 0.05, ^##^ *p* < 0.01 versus the IRI.

The results of lipolytic mRNA expression are shown in Figure 6. The mRNA expression levels of the PPAR-α and ACOX1 (Figure 6A,B) were extremely significantly lower in the IRI group on days post-surgery compared to that in the Sham group (*p* < 0.01), while they were upregulated by ADSCs-Exo and ADSCs (*p* < 0.01 or 0.05 < *p* < 0.01, versus IRI). There were no significant differences in PPAR-α and ACOX1 mRNA expression levels between groups at other points in time. There were no significant differences in the mRNA expression levels of CROT and CPT1A (Figure 6C,D) among the groups at each point in time.

### 2.7. Effect of ADSCs-Exo on the Expression of Protein Related to Lipid Metabolism

**Figure 7:**
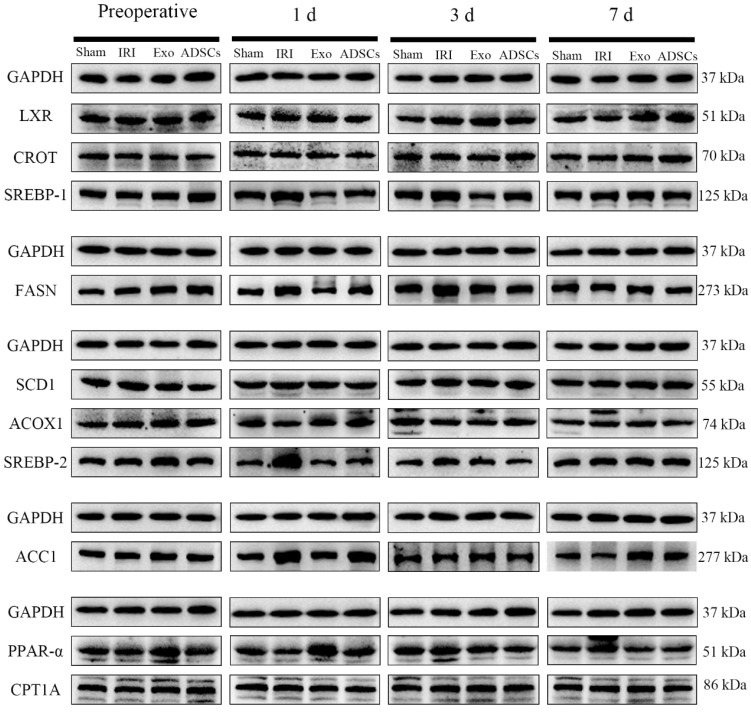
Results of lipid metabolism-related proteins detection.

**Figure 8:**
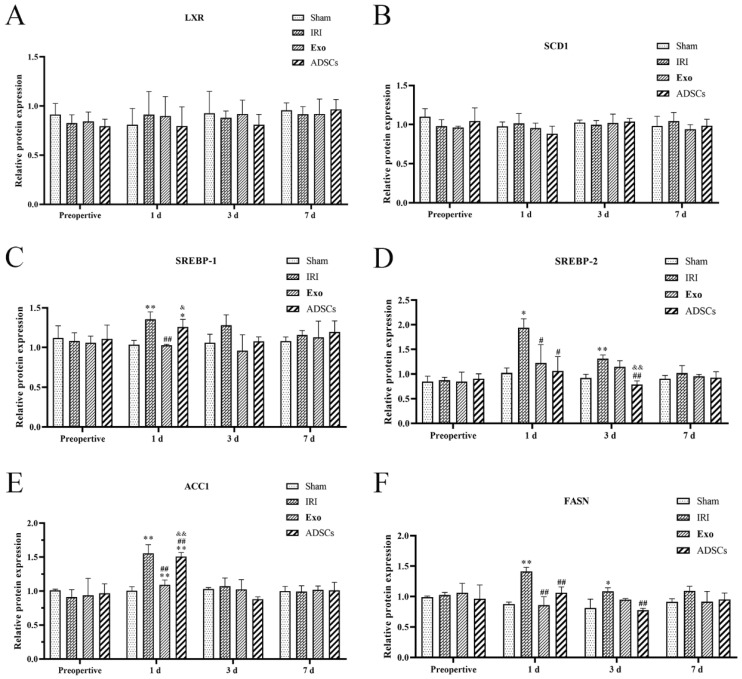
The protein expression of LXR (**A**), SCD1 (**B**), SREBP-1 (**C**), SREBP-2 (**D**), ACC1 (**E**), and FASN (**F**) in the liver. Results are presented as mean ± SD, * 0.01 < *p* < 0.05, ** *p* < 0.01 versus the Sham group. ^#^ 0.01 < *p* < 0.05, ^##^ *p* < 0.01 versus the IRI group. ^&^ 0.01 < *p* < 0.05, ^&&^ *p* < 0.01.

The lipid synthesis protein expression results are shown in Figure 7 and Figure 8. The protein expression levels of LXR and SCD1 (Figure 8A,B) did not differ significantly among the groups at any point in time. The protein expression levels of the SREBP-1, SREBP-2, ACC1, and FASN (Figure 8C–F) were extremely significantly higher in the IRI group on days post-surgery compared to that in the Sham group (*p* < 0.01), while they were downregulated by ADSCs-Exo and/or ADSCs (*p* < 0.01 or 0.01 < *p* < 0.05) versus IRI. The protein expression levels of the SREBP-2, ACC1, and FASN (Figure 5D–F) were extremely significantly higher in the IRI group three days post-surgery compared to that in the Sham group (*p* < 0.01), while they were downregulated by ADSCs-Exo and/or ADSCs (*p* < 0.01 or 0.01 < *p* < 0.05) versus IRI. There were no significant differences in the protein expression levels of SREBP-1, SREBP-2, ACC1, and FASN at other points in time.

**Figure 9:**
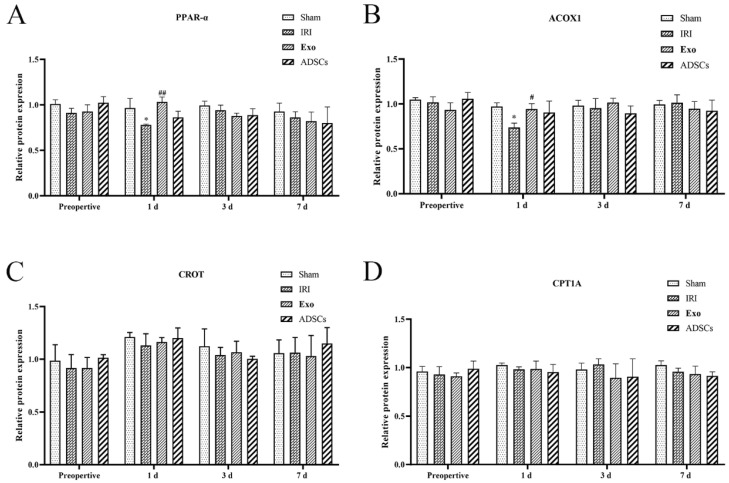
The protein expression of PPAR-α (**A**), ACOX1 (**B**), CROT (**C**), and CPT1A (**D**) in the liver. Results are presented as mean ± SD, * 0.01 < *p* < 0.05 versus the Sham group. ^#^ 0.01 < *p* < 0.05, ^##^ *p* < 0.01 versus the IRI group.

The results of lipolytic protein expression are shown in Figure 7 and Figure 9. The protein expression levels of the PPAR-α and ACOX1 (Figure 9A,B) were significantly lower in the IRI group on days post-surgery compared to that in the Sham group (0.01 < *p* < 0.05), while they were upregulated by ADSCs-Exo (*p* < 0.01 or 0.05 < *p* < 0.01) versus IRI. There were no significant differences in PPAR-α and ACOX1 protein expression levels between groups at other points in time. There were no significant differences in the protein expression levels of CROT and CPT1A (Figure 9).

## 3. Discussion

This study established a minimally invasive laparoscopic partial hepatectomy model combined with IRI in miniature swine to investigate the therapeutic effects of ADSCs-Exo on lipid metabolism abnormalities following liver IRI.

The mechanisms involved in liver IRI are complicated. Liver ischemia causes tissue hypoxia, depletes energy, and results in cell death. Reperfusion restores oxygen, triggering oxidative stress and increasing inflammation, which worsens liver injury [32]. Lipid metabolism also plays a key role in liver damage. Lipid metabolism disorders are intricately linked to the onset and progression of a wide range of liver diseases. The accumulation of lipids can amplify the release of inflammatory factors and lipid peroxides when the liver experiences oxidative stress. In patients with hepatocellular carcinoma undergoing partial liver resection, the prevalence of liver steatosis contributes to an increased risk of postoperative liver failure [33]. This study observed that after liver IRI, liver lipid levels increased while serum lipid levels decreased. Liver histological analysis via oil red O staining and transmission electron microscopy revealed characteristic fatty degeneration within the liver tissue.

MSCs are recognized as a type of versatile stem cell that are ubiquitously distributed across numerous body tissues. A substantial body of evidence derived from animal models and clinical trials has consistently demonstrated the therapeutic potential of MSCs in the treatment of organ damage [34]. In the context of liver disease research, MSCs have received considerable attention due to their potential to alleviate liver injury, enhance liver function, and facilitate hepatocyte regeneration [35]. In the studies focusing on non-alcoholic steatohepatitis (NASH), it has been demonstrated that after the administration of MSCs, there is a significant reduction in liver lipids [36,37]. In the NAFLD model, transplantation of fat-metabolizing hepatocyte-like MSCs has been demonstrated to effectively reduce lipid accumulation, thereby reversing the process of steatosis and ultimately restoring liver function [38,39]. The present experimental study addresses the dysregulation of lipid metabolism following IRI in the liver by transplanting ADSCs. By reversing the aberrant expression of genes and proteins involved in lipid metabolism, ADSCs effectively mitigate excessive fat accumulation within hepatocytes, thereby ameliorating metabolic disorders. This treatment ultimately results in the alleviation of hepatic steatosis and the restoration of functional liver capabilities. The statement is consistent with prior experiments conducted by our research team [40]. In the study of abnormalities of hepatic lipid metabolism, the effects of MSCs on lipid metabolism are beneficial. These effects are achieved by regulating the normal expression of adipose-related genes [41].

Exo are nanoscale (30–150 nm) vesicles secreted by cells and are widely distributed in various bodily fluids [42]. These cargo carriers contain numerous regulatory factors that are crucial for influencing the surrounding environment and communication between cells [43]. Current research shows that MSCs-Exo are essential for the homing process. This process influences cellular proliferation, differentiation, and immune response modulation. Exo have been shown to have a remarkable ability to promote self-repair after cellular injury and aid in tissue regeneration [44]. MSCs-Exo regulates downstream inflammatory genes by delivering miRNAs such as miR-21, miR-146a and miR-181 to promote anti-inflammatory M2 macrophage polarization and inhibit pro-inflammatory M1 macrophages to treat nerve injury [45]. Hypoxic preconditioning of MSCs increases HIF-1α in Exo and promotes bone regeneration in a rabbit model of osteonecrosis [46]. MSC-Exo play a pivotal role in the pathogenesis of numerous pathological conditions. The co-culture of ADSCs-Exo with adult neuroblastoma cells exhibiting high β-amylose expression demonstrated that ADSCs-Exo is capable of transporting substantial quantities of enkephalinase and diminishing intracellular and extracellular β-amylose peptides. These findings suggest that ADSCs-Exo may offer a promising avenue for Alzheimer’s disease treatment [47,48]. Wan et al. [49]. demonstrated that MSCs-Exo protected against acute kidney injury (AKI) by inhibiting focal death in rat and NRK-52E cells. Furthermore, Exo therapy offers the potential to address concerns regarding the safety profile of stem cell applications [50]. An increasing number of studies have demonstrated that MSCs-Exo exert protective effects on the liver. These effects occur by regulating insulin resistance, enhancing lipid metabolism, reducing oxidative stress, and decreasing inflammation [37,51,52]. Studies on the combined effects of MSCs and MSCs-Exo in treating NASH have shown significant reductions in liver fat accumulation and swelling [53]. The therapeutic benefits are due to their capacity to interfere with fatty acid metabolism by downregulating key genes involved in fat synthesis, specifically SREBP-1, SREBP-2, and ACC, as well as inhibiting lipid uptake through CD36. Furthermore, BMSC Exo have been observed to upregulate genes that promote fat oxidation, including PPAR-α and ACOX1 [54].

Despite the encouraging outlook for MSCs transplantation, the majority of ongoing clinical trials are still in the initial phases, typically I or II. The clinical trial failure of autologous and allogeneic MSCs products has been a recurring phenomenon. Moreover, there have been reports indicating an increased risk of tumor formation and cell death following MSCs transplantation [55]. In contrast to MSCs, MSCs-Exo is not capable of self-replication and does not induce tumors of a medically defined nature [56]. With regard to preservation, MSCs are more demanding, and MSCs-Exo can be preserved at −80 °C or in liquid nitrogen. Furthermore, the addition of DMSO is not required, as this better protects the bioactivity of MSCs-Exo [57]. MSCs-Exo lacks MHC antigens and thus presents a minimal risk of alloimmune rejection, which enhances its stability and preservation in vivo. In contrast to MSCs, MSCs-Exo are vesicles that encapsulate a range of biological molecules, including miRNAs, mRNAs, lipids, proteins, genes, and even organelles. Notably, they are capable of crossing the blood–brain barrier [58]. Multiple organ failure is a common occurrence in clinical settings and is typically attributed to mitochondrial dysfunction [58]. MSCs-Exo possess the capacity to encapsulate functional mitochondria, facilitating mitochondrial transfer [58,59]. This phenomenon, coupled with the inherent limitations of clinical applications involving MSCs [60], underscores the potential value of further research and applications of MSCs-Exo. Engineered Exo improve therapeutic efficacy through bioengineering techniques that modify Exo or donor cells in a more precise manner. This results in Exo having a longer circulating half-life and greater targeting ability, as well as reducing drug systemic distribution and adverse effects [61]. In a mouse breast cancer model, Gomari et al. [62]. demonstrated that MSCs-Exo-delivered adriamycin markedly diminished tumor growth. Exo are biocompatible and can effectively mitigate the toxicity and adverse effects of chemotherapeutic drugs. It has been demonstrated that large nucleic acid substances, including mRNA and DNA, can be loaded into Exo through transfection or electroporation methods [63]. This allows for their delivery to tumor cells, where they can inhibit cellular activity and promote apoptosis, ultimately improving the survival rate of mice. Concurrently, the utilization of exosome advantages in conjunction with tissue engineering materials can facilitate enhanced drug delivery efficiency. Han et al. [64]. developed a hybrid microneedle array patch comprising MSCs-Exo and hydrogel. This patch was employed to facilitate the local release of Exo in situ following spinal cord surgery. This approach not only enhanced the efficacy of a single administration but also circumvented the potential complications associated with repeated local injection of Exo. In a study conducted by Hu et al. [65]., Exo-coated eluting stents were found to effectively accelerate re-endothelialization and reduce restenosis within the stent. Qi et al. [66]. modified the transferrin receptor of blood-derived exosomes with magnetic nanoparticles and subsequently utilized external magnets to direct the exosomes to mouse tumors, thereby creating targeted drug delivery vehicles for cancer therapy. The aforementioned evidence collectively suggests that MSC-Exo has considerable potential as a cell-free therapeutic agent. In conclusion, MSC-Exo demonstrate considerable promise as a cell-free therapeutic modality.

Numerous studies [50,67,68] have shown that MSCs-Exo can transport various components and are effective in treating liver metabolic diseases. Although there is extensive research, most studies have focused on small animal models. Investigations involving larger animals are still in the early stages. As a large model animal, the miniature swine is very similar to humans in terms of anatomical structure, organ size, and physiology [69], as well as biochemical characteristics and genome [70], which can compensate for the shortcomings of rodent models that lack the ability to study some human diseases. In a study related to hepatic lipid abnormalities, Lee et al. [71]. established the first large animal model of diet-induced steatohepatitis by feeding high-fat diets to minipigs for 24 weeks, which showed characteristics very close to those of the metabolic syndrome of NAFLD in humans. The knockout of the leptin gene in Chinese experimental miniature swine was achieved using zinc finger nuclease gene editing technology [72]. The resulting phenotype exhibited a developmental pattern of non-alcoholic liver injury that was consistent with that observed in the obese population. In porcine-primate liver xenotransplantation [73], the porcine liver is also capable of sustaining essential lipid metabolism over time in non-primates. It can be demonstrated that the pig is a more appropriate model for the study of abnormalities in human hepatic lipid metabolism. This study demonstrated the potential of Exo to ameliorate lipid abnormalities and liver IRI in large animals using miniature swine as experimental subjects. These findings provide a basis for future studies of liver dyslipidaemia in large animals and, from a comparative medicine perspective, provide new insights into the treatment of dyslipidaemia caused by liver injury in humans.

There are some limitations in this study, which only broadly investigated the effect of Exo on lipid metabolism-related indexes after IRI in miniature swine livers, and did not explore in depth the specific role of Exo in regulating lipid metabolism in these cell organelles, and the specific pathways and targets were not clarified. In addition, what kind of substances in Exo play a role also needs to be further explored.

## 4. Materials and Methods

### 4.1. Experimental Animals

Following approval from the Institutional Animal Care and Use Committee at Northeast Agricultural University, we used twenty-four healthy Bama miniature swine, aged 3 to 4 months and weighing between 20 and 30 kg, with free access to food and water in this experiment.

### 4.2. Isolation and Culture of ADSCs

Miniature swine were anesthetized using standard procedures, and inguinal fat was collected aseptically. Fascial vessels were excised in a sterile chamber. The fat was then cut into small pieces and digested with 0.1% collagenase type I in a 37 °C water bath. After digestion, the mixture was centrifuged at 1500 rpm for 10 min. The precipitate was added to a complete medium consisting of low-sugar DMEM, 10% FBS, 1% penicillin-streptomycin, and 1% glutamine to complete digestion, and then filtered through a 200-mesh copper sieve. The filtrate was centrifuged, then resuspended in complete medium and inoculated into flasks at a density of 2.5 to 3 × 10^4^ cells/cm^2^. The medium was changed after 24 to 48 h to eliminate unattached cells. Cells were passaged once they reached 70 to 80% confluence.

### 4.3. Characterization of ADSCs

Single cell suspensions were prepared following the instructions for the antibodies: CD29 (Abcam, ab21845, Cambridge, UK), CD44 (Abcam, ab95138), CD90 (Abcam, ab124527), and CD11b (Biolegend, 301329, San Diego, CA, USA). Flow cytometry was used to detect the positively stained ADSCs, and the analysis was performed with FACSD software (FACSDiva Version 6.1.3, BD, New York, NY, USA). The adipogenic and hepatogenic differentiation of ADSCs from 3 to 5 generations was conducted according to the differentiation kit instructions.

### 4.4. Isolation and Purification of ADSCs-Exo

Cells were cultured until they reached 80% confluence after 3 to 5 generations. The medium was then removed, and the cells were washed three times with PBS. Finally, a serum-free medium was added, and the cells were placed in a 37 °C, 5% CO_2_ incubator for further culture. The cell culture supernatant was collected after 36 to 48 h. The cells were sequentially centrifuged at 300× *g* for 15 min to remove dead cells, and then at 2000× *g* for 20 min to remove cell debris, all at 4 °C. The supernatant was filtered through a 0.22 μm filter to eliminate larger extracellular vesicles. The final ADSCs-Exo, which appeared as a tan precipitate, were suspended in sterile PBS and stored at −80 °C for future use.

### 4.5. Identification of ADSC-Exo

The nanoparticle tracking analysis (NTA) from Particle Metrix (Inning am Ammersee, Germany) was used to determine the size and number of ADSC-Exo. Subsequently, the morphology of the Exosomes was examined using transmission electron microscopy (TEM) from Hitachi (Tokyo, Japan), employing the phosphotungstic acid negative staining method. Western blot analysis was conducted to identify the characteristic Exosomal antigens CD63, CD81, and TSG101.

### 4.6. Surgical and Experimental Design

Twenty-four healthy Bama miniature swine, aged 3 to 4 months and weighing between 20 and 30 kg, were randomly assigned to four groups: Sham, IRI, Exo, and ADSCs. Liver IRI combined with partial resection was performed as previously described. Briefly, the miniature swine were fasted for 24 h and hydrated for 12 h. Anesthesia was maintained by tracheal intubation with isoflurane after induction with propofol. The abdomen was routinely sterilized, and a four-cannula laparoscopic surgical access with 10 mmHg pneumoperitoneum was established. A homemade tourniquet was used to block blood flow to the right half of the liver. After 1 h, the tourniquet was removed to restore blood supply. The blood flow to the left half of the liver was blocked and resected in the same manner, and finally, the corresponding therapeutic substances were slowly injected through the portal vein. The Exo group received an injection with 5 × 10^9^ ADSC-Exo per kg dissolved in 5 mL of sterile PBS, while the ADSCs group was injected with 1 × 10^6^ ADSCs per kg, also dissolved in 5 mL of sterile PBS. The Sham and IRI groups received the same volume of sterile PBS. Following the injection of the therapeutic substance and confirmation of no bleeding, the abdominal cavity was rinsed with saline, and the resected left half of the liver was placed in a specimen bag for removal. Gas was drained from the abdominal cavity, and the trocar opening was routinely sutured. Anesthesia was switched off, and the animals were managed postoperatively. Blood was collected from the anterior vena cava. Liver tissue specimens were obtained using minimally invasive laparoscopic techniques on the preoperative day, as well as on days 1, 3, and 7, postoperatively.

### 4.7. Ultrastructural Analysis

Fresh liver tissue samples were first cut into 1 mm^3^ pieces, then pre-fixed in 2.5% glutaraldehyde, post-fixed in 1% osmium, embedded in neutral resin, and finally sectioned into ultrathin slices. The specimens were stained with uranyl acetate and lead citrate, then examined under a transmission electron microscope to observe lipid droplets in the hepatocytes.

### 4.8. Oil Red O Stain

Frozen liver tissues were embedded in an OTC medium and sliced into 8 μm sections using a frozen sectioning machine. The sections were stained with oil red O and subsequently re-stained with hematoxylin. Under the microscope, the lipid droplets appeared red.

### 4.9. Measurement of HDL, LDL, TG and CHOL

Blood samples were centrifuged at 3500 rpm for 15 min. Serum levels of HDL, LDL, TG, and CHOL were then measured using a BioTek blood biochemistry analyzer (TBA-2000FR, Canon Medical. Systems Corporation, Tochigi, Japan).

A 10% liver tissue homogenate was prepared using cold saline. This homogenate was processed according to the instructions of the HDL(Jiangcheng, Nanjing, China), LDL, TG, and CHOL kits. Measurements were taken at the appropriate wavelengths using an enzyme marker.

### 4.10. RNA Extraction and Real-Time Quantitative Polymerase Chain Reaction

Total RNA was extracted from liver tissues using Trizol (Invitrogen, Carlsbad, CA, USA). The RNA was then quantified and reverse transcribed into cDNA. The target gene was amplified using a three-step RT-PCR process in the LightCycler 480 (Roche, Basel, Switzerland) with the SYBR Green I reaction mix (Vazyme, Nanjing, China). The relative fold change in gene expression was calculated using the 2^−ΔΔCt^ method and normalized to β-actin levels. Primers were synthesized by UW Genetics, and their sequences are provided in Table 1.

### 4.11. Western Blotting

The total proteins were extracted from liver tissues and analyzed using Western Blotting, following the methods outlined in reference [74]. The nitrocellulose membrane was incubated with LXR (1:1000, Bioss, bs-18451R, Beijing, China), CROT (1:1000, Bioss, bs-6365R), SREBP-1 (1:1000, Immunoway, YT6055, Plano, TX, USA), FASN (1:1000, ABclonal, A21182, Wuhan, China), SCD1 (1:1000, Bioss, bs-3787R), ACOX1 (1:1000, Bioss, bs-5021R), SREBP-2 (1:500, Santa Cruz, sc-13552, Santa Cruz, CA, USA), ACC1 (1:1000, ABclonal, A19267), PPAR-α (1:1000, Bioss, bs-3614R), CPT1A (1:1000, Bioss, bs-23779R), and GAPDH (1:50,000, ABclonal, A19056) at 4 °C. Next, the membranes were incubated at room temperature for 2 h with the appropriate horseradish peroxidase (HRP)-conjugated secondary antibodies (Bioss, Beijing, China). Protein bands were developed using ECL (meilunbio, Dalian, China) with the Tanon 5200 system (Tanon Science & Technology Co., Ltd., Shanghai, China). The average optical density of the bands was then calculated using ImageJ (1.53, NIH, Bethesda, MD, USA).

### 4.12. Statistical Analysis

Statistical analysis was performed using Graphpad prism 8.0. All data were expressed as mean ± standard deviation. One-way ANOVA and LSD tests were used to compare the groups, and a *p*-value less than 0.05 was considered statistically significant.

## 5. Conclusions

ADSCs-Exo have shown the potential to improve lipid metabolism disorders following liver ischemia-reperfusion injury in miniature swine. Our research findings support the development of future medical treatments for lipid metabolism disorders in larger animals and, eventually, in humans in the area of liver health.

## Figures and Tables

**Table 1:** Gene-specific primers used for RT-qPCR.

Gene	Forward Primer Sequence (5′-3′)	Reverse Primer Sequence (5′-3′)
*β-actin*	TCTGGCACCACACCTTCT	TGATCTGGGTCATCTTCTCAC
*LXR*	GTTGCACATGGCCTGGTCAC	CTCCACTGCAGAGTCAGGAGA
*CROT*	AGCTTCACCCTGATGCGTTT	GCCGCTCAGAAAGACTGGT
*SREBP-1*	CAGCTCCATTGACAAGGCCA	GCACCCCATCTACACTACGC
*FASN*	TGGATCACTGCATAGACGGC	TGGTACACCTTCCCGCTTG
*SCD1*	TCATTGGGAGCTGTGGGTGAG	ACAGGGGCTTTCCCAGAAGAT
*ACOX1*	GAACCAGGACCTACAGAAGGAG	TCCTCGCTGCACAAAGTTTTTA
*SREBP-2*	CTCGGTTTCGGCAGACCAT	TGGTGAAGGAACCAGCTCTT
*ACC1*	TGGCTAAACCTCTGGAGTTGAA	CTGCCATCTTAATGTATTCAGCGT
*PPAR-α*	TTTCCACAAGTGCCTCTCGG	GTGTATGACGAAAGGCGGGT
*CPT1A*	ATGTACGCCAAGATCGACCC	CCACCAGTCGCTCACGTAAT

## Data Availability

The data used to support the findings of this study are available from the corresponding author upon request.
